# Comparative association between NAFLD and MAFLD with cardiovascular events and mortality: Evidence from observational studies

**DOI:** 10.1371/journal.pone.0312650

**Published:** 2025-06-13

**Authors:** Taoli Fu, Jitao Ling, Huilei Zhao, Kaixin Lin, You Deng, Miao Zhang, Xinrui Qi, Peng Yu, Weichun Lin, Xiao Liu

**Affiliations:** 1 Department of Endocrinology, Nanchang People’s Hospital, Nanchang, Jiangxi, China; 2 Department of Endocrinology and Metabolism, The Second Affiliated Hospital, Jiangxi Medical College, Nanchang University, Nanchang, Jiangxi, China; 3 Department of General Surgery (Colorectal Surgery), the Sixth Affiliated Hospital, Sun Yat-sen University, Guangzhou, China; 4 Department of Gastroenterology, the Third Affiliated Hospital, Sun Yat-sen University, Guangzhou, China; Universita degli Studi della Campania Luigi Vanvitelli Scuola di Medicina e Chirurgia, ITALY

## Abstract

**Background:**

Nonalcoholic fatty liver disease (NAFLD) and metabolic dysfunction-associated fatty liver disease (MAFLD) are increasingly recognized as multisystemic conditions with significant extrahepatic manifestations. Although both diseases have been linked to cardiovascular events and mortality, the strength of these associations remains controversial. This review aims to compare the risks of cardiovascular disease (CVD) events in individuals with NAFLD and MAFLD.

**Methods:**

The PubMed, Embase, and Cochrane Library databases were searched to identify studies investigating the risk of CVD, CVD death, and all-cause death associated with NAFLD and MAFLD through April 28th, 2024. Data extraction and study quality assessment were performed using the Newcastle-Ottawa Scale. Statistical analysis utilized random-effects models. The quality of evidence was assessed using the Grading of Recommendations Assessment, Development, and Evaluation (GRADE) methodology.

**Results:**

Eleven cohort studies with 11,995,994 patients were included. Both NAFLD and MAFLD were associated with an increased risk of cardiovascular events (NAFLD group vs. non-NAFLD group: HR = 1.30, 95% confidence interval (CI): 1.13–1.49, I^2^ = 91%; MAFLD group vs. non-MAFLD group: HR = 1.54, 95% CI: 1.32–1.81, I^2^ = 92%) and all-cause death (NAFLD group vs. non-NAFLD group: HR = 1.18, 95% CI: 1.04–1.33, I^2^ = 55%; MAFLD group vs. non-MAFLD group: HR = 1.30, 95% CI: 1.20–1.40, I^2^ = 0%). The MAFLD-only group had a stronger association with cardiovascular death than the NAFLD-only group (MAFLD-only group vs neither MAFLD nor NAFLD group: HR = 1.46, 95% CI: 1.40–1.51, I^2^ = 0%; NAFLD-only group vs neither MAFLD nor NAFLD group: HR = 1.10, 95% CI: 0.95–1.28, I^2^ = 0%, P for interaction < 0.01). The GRADE assessment revealed low certainty for cardiovascular disease (CVD) (NAFLD only group vs. neither NAFLD nor MAFLD group), cardiovascular death (NAFLD only group vs. neither NAFLD nor MAFLD group), and all-cause death (MAFLD group vs non-MAFLD group), and very low certainty for other results.

**Conclusion:**

MAFLD and NAFLD are both associated with cardiovascular events and all-cause mortality. However, the odds of cardiovascular death seems to be greater in patients with MAFLD than in those with NAFLD, suggesting that the use of MAFLD criteria may identify more at-risk individuals.

**PROSPERO registration:**

CD42022361164.

## Introduction

Nonalcoholic fatty liver disease (NAFLD) is a liver problem that affects individuals with little to no alcohol consumption. Its global prevalence in adults is approximately 25% [1]. In recent years, NAFLD has become one of the most common chronic liver diseases, and it leads to liver cancer and cirrhosis [2]. In 2017, there were 2.14 million liver-related deaths, representing an 11.4% increase since 2012 [3]. Patients with NAFLD often present with an abnormal buildup of fat in their liver tissue, exceeding normal levels. Currently, no specific medications are available to treat NAFLD. Two major types of tests used to diagnose NAFLD are blood tests and imaging tests to visualize the appearance of the liver, such as computed tomographic scans, ultrasound, and magnetic resonance imaging. In recent years, more advanced tests that quantify the amount of fat in the liver, such as transient elastography, have been proposed. During diagnosis, other potential causes of liver disease or steatosis must be excluded [4]. Nevertheless, although the presence of metabolic risk factors is strongly associated with NAFLD, the diagnosis of NAFLD itself does not strictly depend on the presence of these factors [5].

Recent evidence has shown that NAFLD can also affect extrahepatic organs and metabolic pathways [6,7]. Studies have reported that NAFLD is a risk factor for various chronic diseases, especially metabolic syndrome and cardiovascular diseases (CVDs) [8–11]. In 2020, two international panels of experts proposed a new definition for metabolic dysfunction-associated fatty liver disease (MAFLD) to provide “positive criteria” for diagnosis [12]. This new definition of MAFLD represents the hepatic manifestation of systemic metabolic dysregulation and removes the concept of alcohol involvement [13]; however, this new diagnostic criterion is still debatable. Additionally, several studies have highlighted the role of fatty liver in predicting the risk of CVD and other chronic diseases. Cardiovascular diseases, which caused approximately 17.8 million deaths globally in 2017 [14], are the most common noncommunicable diseases. Several studies have shown that patients with NAFLD have a greater risk of developing CVD and experiencing CVD-related mortality, and all-cause mortality [15–17].

Although several studies have compared the association between NAFLD and/or MAFLD and CVD, the results are still controversial [18–20]. Additionally, it is important to note that a small subset of the population meets only one of the diagnostic criteria; as predicted, a subset meets only the MAFLD but not the NAFLD definition, which has more metabolic risk factors. Additionally, the population meeting only the NAFLD definition but not the MAFLD definition has relatively fewer metabolic risk factors. Due to the differences between the two diagnostic terms, the comparison of outcomes in this small population subset may provide valuable insights into understanding the implications of these two conditions. Thus, the objective of this systematic review and meta-analysis was to compare the associations of NAFLD and MAFLD with the risk of cardiovascular events, cardiovascular mortality, and all-cause mortality. We hypothesize that individuals with MAFLD have a greater risk of cardiovascular events than those with NAFLD.

## Methods

This meta-analysis was reported following PRISMA guidelines (http://www.prisma-statement.org; S1 Table in S1 File). The protocol was registered with PROSPERO (International Prospective Register of Systematic Reviews. https://www.crd.york.ac.uk/PROSPERO/-registration number CD42022361164).

### Literature search

Two authors (X. L. and W-C. L.) independently conducted the analysis through April 28, 2024 by consulting the PubMed, Embase, and Cochrane Library databases. The following keywords and medical subject headings were applied: “MAFLD”, “NAFLD”, “cardiovascular”, “death”, and “mortality”. The detailed search process is shown in S5 Table in S1 File. Any disagreements were resolved by consulting a third author (P. Y.).

### Study selection

The inclusion and exclusion criteria were set based on the PICOS framework (population, intervention, comparison, outcome, and study design). The inclusion criteria were as follows: (1) participants: adults aged >18 years; (2) exposure and comparator: subjects with NAFLD vs. subjects without NAFLD; subjects with MAFLD vs. subjects without MAFLD; subjects with NAFLD-only vs. subjects with neither MAFLD nor NAFLD; subjects with MAFLD-only vs. subjects with neither MAFLD nor NAFLD; (3) outcomes: cardiovascular events, cardiovascular mortality, and all-cause mortality were determined, and the associated adjusted relative risk (RR)/hazard ratio (HR)/odds ratio (OR) with corresponding 95% confidence intervals (CIs) or other measures that could be used to compute these values, were reported; (4) types of studies: designed as cohort studies or case-control studies. If multiple studies used the same population, then the most rigorously designed study was chosen that reported adjusted relative risks, hazard ratios, or odds ratios for the outcomes of interest (cardiovascular events, cardiovascular mortality, and all-cause mortality); (5) language of the study: without language restriction. Conference abstracts, editorials, animal studies, and reviews or studies with unavailable data were excluded from this analysis.

### Data extraction and quality assessment

For each included article, the following key information was extracted: first author, publication year, study region, participant characteristics (sample size, sex ratio, age, country of origin), duration of follow-up, diagnostic technique for hepatic steatosis, data source, and the associated adjusted risk ratio (RR), hazard ratio (HR), or odds ratio (OR) with corresponding 95% confidence intervals (CIs). The Newcastle‒Ottawa Scale (NOS) was used to assess study quality, with scores > 6 indicating high quality. [21].

### Statistical analysis

All statistical analyses were performed using Stata software (version 16.0, Stata LP, University of Texas Station, USA) and RevMan (Review Manager [RevMan], version 5.4, Cochrane Collaboration) software. The presence of heterogeneity was measured by the Q test (*P* < 0.10 was considered statistically significant [22]. I^2^ was considered as the inconsistency among studies. Considering the potential heterogeneity, the random-effects model was applied. A sensitivity analysis was performed using a fixed model when the heterogeneity was not significant. Funnel plots and Egger’s and Begg’s tests were used to detect publication bias. A *P*- value for the interaction of <0.05 indicated a statistically significant subgroup effect. A two-tailed *P* < 0.05 was considered statistically significant in other analyses.

### Quality of evidence

The quality and strength of the evidence for each outcome were assessed according to the Grading of Recommendations Assessment, Development, and Evaluation (GRADE) method [23,24]. GRADE profiler software was used to provide evidence profile tables. Two authors (X. L. and W-C. L.) assessed the quality of the evidence for each outcome separately.

## Results

### Study selection

**Fig 1:**
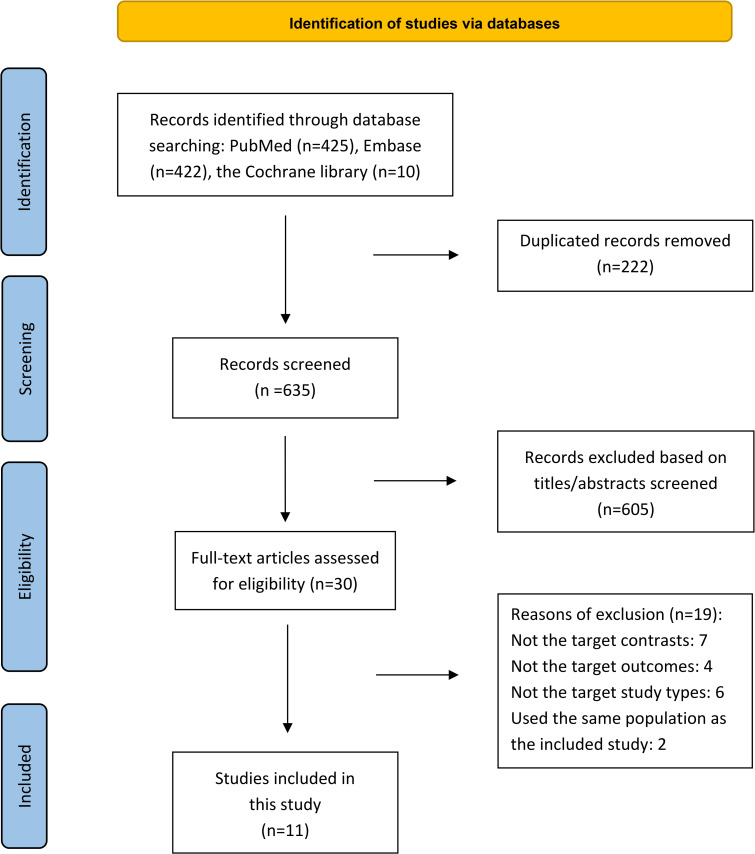
Flowchart of the search and the selection process of the meta-analysis of the comparative association between NAFLD and MAFLD with cardiovascular events and mortality. MAFLD: Metabolic-associated fatty liver disease; NAFLD: Non-alcoholic fatty liver disease.

The flow chart shows an overview of the search and selection process (Fig 1). An initial search of 857 articles (PubMed: 425; Embase: 422; Cochrane Library: 10) was performed according to the search protocol. After removing duplicates and screening titles and abstracts, 30 articles were selected for whole-paper evaluation, 19 of which were further excluded for the following reasons: (1) did not include target control (n = 7); (2) did not include target outcomes (n = 4); (3) certain publication types for which data were not available (three were reviews; one was an editorial; one was a letter; one was a survey) (n = 6); and (4) same population (n = 2). (S2 Table in S1 File). Finally, eleven articles, including 11,995,994 people, were included in this meta-analysis.

### Study characteristics and quality

The basic characteristics of the included studies are summarized in Table 1. A total of eleven cohort studies [25–35] were eligible for inclusion, with 11,995,994 subjects whose median follow-up time ranged from 4 to 27 years. All these studies were published in 2021–2024. The number of study samples ranged from 2,985–8,962,813, with a mean age between 39 and 62 years and the proportion of men ranged from 37% to 63%. Six articles detected fatty liver by ultrasound examination [26,28,30,33,34,36], three articles defined fatty liver by the fatty liver index [27,29,31], and two articles used the K-NAFLD score [25,32]; the diagnostic criteria are shown in S4 Table in S1 File. Age was the variable for which all the observational studies adjusted their findings. All the observational studies had NOS scores > 6, indicating that all the studies were of acceptable quality (S3 Table in S1 File).

**Table 1:** Characteristics of included studies in this meta-analysis of the comparative association between NAFLD and MAFLD with cardiovascular events, and mortality.

References (first author, year, country/region)	Type of study	Sample size	NAFLD/ NAFLD+MAFLD-	MAFLD/ NAFLD-MAFLD+	Age (year)/male (%)	Duration of follow-up	Outcomes reported	HR (95%CI)	Adjustments for confounders
Han, 2024, Korea	RC	26,734	7171/NA	9120/NA	49 years (mean)/43	110.6 months (mean)	All-cause death: Non-NAFLD NAFLD Non-MAFLD MAFLD	Ref 1.28(1.14, 1.44); Ref 1.34(1.21, 1.50)	Age, sex, alcohol drink, smoking, low social economic status, regular exercise, body mass index, hypertension, diabetes, viral hepatitis, dyslipidemia, chronic kidney disease, prior history of cardiovascular disease, and malignancy.
Jeong, 2021, Korea	RC	333,389	41,915/ NA	23,190/ NA	57 years (mean)/54	5.5 years (mean)	CVD: Non-NAFLD NAFLD Non-MAFLD MAFLD	Ref 1.55(1.44, 1.65); Ref 1.71(1.22, 2.41)	Age, sex, insurance premium, BMI, smoking, alcohol consumption, physical activity, ALT, Charlson Comorbidity Index.
Kim, 2021, America	RC	7,761	2,438/394	2,256/212	41 years (mean)/50	23.0 years (median)	CV death: Neither NAFLD nor MAFLD NAFLD-only MAFLD-only All-cause death: Neither NAFLD nor MAFLD NAFLD-only MAFLD-only	Ref 0.62(0.2, 1.92), 0.98(0.46, 2.08); Ref 0.94(0.6, 1.46), 1.66(1.19, 2.32).	Age, sex, race/ethnicity, education, marital status, smoking status, alanine aminotransferase, and sedentary lifestyle, body mass index, diabetes, hypertension, fasting triglycerides, high-density lipoprotein cholesterol, waist circumference and CRP.
Kim, 2023, South Korea	RC	394,835	NA/5,979	NA/16,952	40 years (mean)/55	5.7 years (median)	CV death: Neither NAFLD nor MAFLD NAFLD-only MAFLD-only All-cause death: Neither NAFLD nor MAFLD NAFLD-only MAFLD-only	Ref 0.87(0.28, 2.74), 1.18(0.77, 1.83); Ref 0.98(0.66,1.46), 0.96(0.80, 1.16).	Age, daily alcohol consumption, regular physical activity, smoking status, total cholesterol, and statin use.
Kim, 2024, America	RC	7,811	2,423/NA	2,244/NA	39 years (mean)/47	27.1 years (median)	CV death: Non-NAFLD NAFLD Non-MAFLD MAFLD All-cause death: Non-NAFLD NAFLD Non-MAFLD MAFLD	Ref 0.85(0.72, 1.00); Ref 0.95(0.80, 1.12); Ref 1.08(0.97, 1.20); Ref 1.23(1.09, 1.38);	Age, sex, race/ethnicity, education, marital status, smoking status, alcohol consumption, sedentary lifestyle, body mass index, diabetes, hypertension and total cholesterol.
Lee, 2021, Korea	RC	8,962,813	2,461,072/54,896	3279143/948,323	50 years (mean)/48	10.1 years (median)	CVD: Non-NAFLD NAFLD Non-MAFLD MAFLD Neither NAFLD nor MAFLD NAFLD-only MAFLD-only CV death: Neither NAFLD nor MAFLD NAFLD-only MAFLD-only	Ref 1.41(1.4, 1.43); Ref 1.52(1.51, 1.54); Ref 1.09(1.03, 1.15), 7.2(2.4, 21.50); Ref 1.12(0.96, 1.30), 1.46(1.41, 1.52).	Age, sex, household income quartile, residential area, Carlson Comorbidity Index, tobacco use, exercise frequency, and estimated glomerular filtration rate.
Liang, 2022, China	RC	6,395	2,545/ NA	2,950/ NA	62 years (mean)/42	4.6 years (mean)	CVD: Non-NAFLD NAFLD Non-MAFLD MAFLD	Ref 1.48(1.17, 1.88); Ref 1.44(1.15, 1.81)	Sex, age, educational background, smoking status, and leisure-time exercise at baseline.
Moon, 2021, Korea	PC	8919	1,142/ NA	1,509/ NA	52 years (mean)/48	15.7 years (mean)	CVD: Non-NAFLD NAFLD Non-MAFLD MAFLD All-cause death: Non-NAFLD NAFLD Non-MAFLD MAFLD	Ref 0.99(0.82, 1.21); Ref 1.07(0.89, 1.3); Ref 1.2(0.94, 1.53); Ref 1.36(1.08, 1.73)	Age, sex, BMI, smoking, alcohol intake, T2DM, hypertension, dyslipidaemia, chronic kidney disease, viral hepatitis, plasma CRP.
Niriella, 2021, Sri Lanka	PC	2,985	940/30	990/57	53 years (median)/37	7.0 years (median)	CVD: Non-NAFLD NAFLD Non-MAFLD MAFLD Neither NAFLD nor MAFLD NAFLD-only MAFLD-only	Ref 3.7(1.30, 10.30); Ref 4.2(1.50, 11.5); Ref 1.9(0.25, 14.8); 7.2(2.4, 21.5).	Age, sex.
Yoneda, 2021, Japan	RC	1,542,688	142,158/ NA	237,242/ NA	46 years (mean)/63	4.0 years (median)	CVD: Non-NAFLD NAFLD Non-MAFLD MAFLD	Ref 1.02(0.92, 1.14); Ref 1.89(1.78, 2.01)	Age, sex, smoking habit, LDL, and statin use.
Yoo, 2023, Korea	RC	701,664	157,548/NA	177,731/NA	40 years/53	8.8 years (median)	CV death: Non-NAFLD NAFLD Non-MAFLD MAFLD	Ref 1.07(0.95, 1.21); Ref 1.14(1.02, 1.28)	Age, sex, education, smoking, regularexercise (3times/week), and plasma LDL-cholesterol.

Abbreviations: HR, hazard ratios; CI, confidence interval; NA, Nonavailable; Ref, reference; FLI, Fatty Liver Index; CVD, cardiovascular disease; CV death, cardiovascular death; NAFLD, Non-alcoholic fatty liver disease; MAFLD, metabolic dysfunction-associated fatty liver disease; NAFLD only, fatty liver disease excluded from MAFLD but captured by the NAFLD; MAFLD only, fatty liver disease excluded from NAFLD but captured by the MAFLD; BMI, Body Mass Index; ALT, alanine aminotransferase; T2DM, type 2 diabetes mellitus; CRP, C-reactive protein; LDL, low density lipoprotein. PC, Prospective cohort study; RC, Retrospective cohort study

### Cardiovascular events between NAFLD and MAFLD

**Fig 2:**
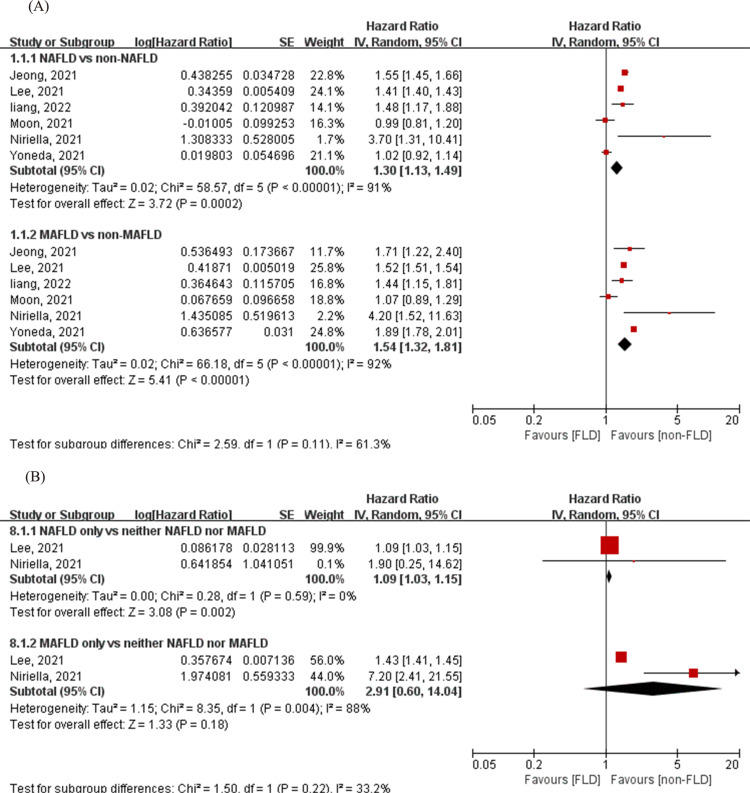
Forest plot for the association between NAFLD/MAFLD and CVD. (A) NAFLD vs. non-NAFLD and MAFLD vs. non-MAFLD. (B) NAFLD-only vs. neither NAFLD nor MAFLD and MAFLD-only vs neither NAFLD nor MAFLD. The diamond indicates the pooled estimate. Red boxes are relative to the study size, and the black vertical lines indicate the 95% CIs around the effect size estimate. MAFLD: Metabolic-associated fatty liver disease; NAFLD: Non-alcoholic fatty liver disease.

Six studies, including 10,857,189 participants, were used to analyze the comparative relationships between NAFLD and MAFLD with cardiovascular events [25,27–31]. Patients with NAFLD or MAFLD both presented significant increases in cardiovascular events (NAFLD vs. non-NAFLD: HR = 1.30, 95% CI: 1.13–1.49, Q- statistic -*P *< 0.1, I^2^ = 91%; MAFLD vs. non-MAFLD: HR = 1.54, 95% CI: 1.32–1.81, Q- statistic -*P* < 0.1, I^2^ = 92%, *P* for interaction = 0.11) (Fig 2). There were only two articles [27,30] with the NAFLD-only group (those excluded from MAFLD but captured by NAFLD) (n = 54,926) and the MAFLD-only group (those excluded from NAFLD but captured by MAFLD) (n = 948,323). The HR for cardiovascular events in the NAFLD-only group was 1.09 (95% CI: 1.03–1.15) with low heterogeneity (Q -statistic -*P* = 0.59, I^2^ = 0%), and 2.91 (95% CI: 0.60–14.04) in the MAFLD-only group with high heterogeneity (Q -statistic -*P* < 0.1, I^2^ = 88%) with a P value of 0.22 for the interaction, compared to the neither NAFLD nor MAFLD group (Fig 2).

### Cardiovascular mortality between NAFLD and MAFLD

**Fig 3:**
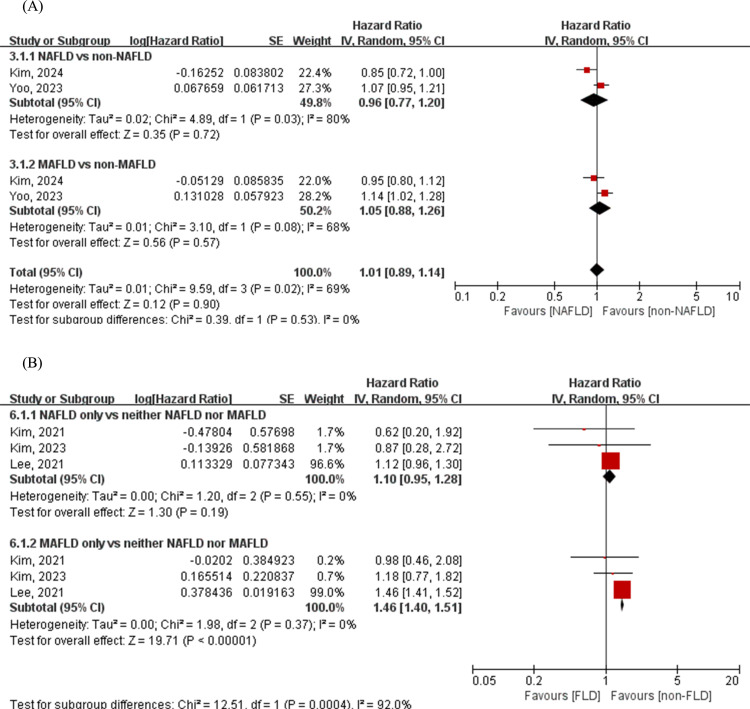
Forest plot for the association between NAFLD/MAFLD and CV death. (A) NAFLD vs. non-NAFLD and MAFLD vs. non-MAFLD; (B) NAFLD-only vs. neither NAFLD nor MAFLD and MAFLD-only vs. neither NAFLD nor MAFLD. The diamond indicates the pooled estimate. Red boxes are relative to the study size, and the black vertical lines indicate the 95% CIs around the effect size estimate. MAFLD: Metabolic-associated fatty liver disease; NAFLD: Non-alcoholic fatty liver disease.

Based on the results of the two included studies [26,35], the relationships between cardiovascular mortality and NAFLD (n = 159,971) (HR = 0.96, 95% CI: 0.77–1.20) or MAFLD (n = 179,975) (HR = 1.05, 95% CI: 0.88–1.26) was not statistically significant. Furthermore, analysis of three cohort studies [26,27,33] revealed that the MAFLD-only group (n = 965,487) was associated with an increased risk of cardiovascular mortality (HR = 1.46, 95% CI: 1.40–1.51), Q -statistic -*P* = 0.37, I^2^ = 0%) whereas there was no statistically significant relationship between the NAFLD-only group (n = 61,269) and cardiovascular death (HR = 1.10, 95% CI: 0.95–1.28), Q -statistic -*P* = 0.55, I^2^ = 0%). Moreover, the association with cardiovascular death was stronger in the MAFLD-only group than in the NAFLD-only group (*P* for interaction < 0.01) (Fig 3).

### All-cause mortality between NAFLD and MAFLD

**Fig 4:**
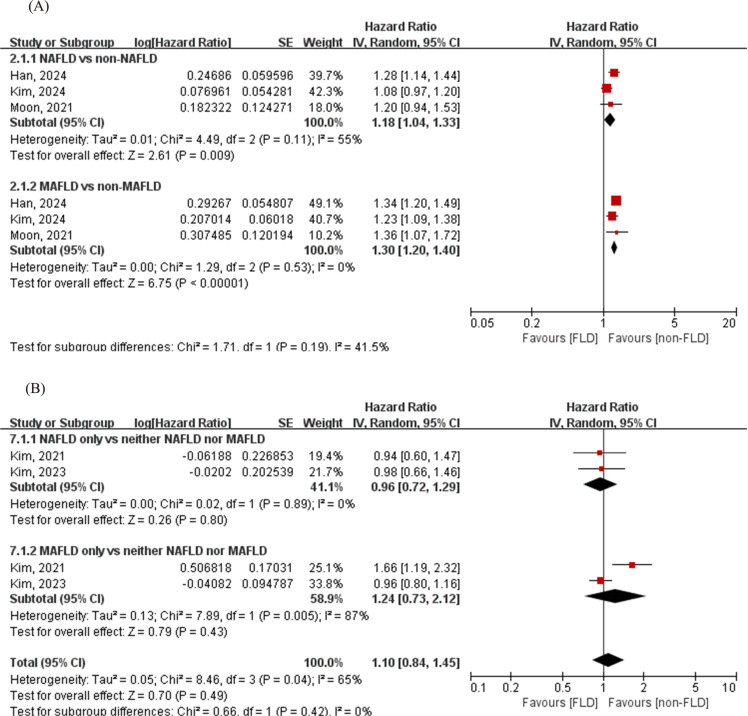
Forest plot for the association between NAFLD/MAFLD and all-cause death. (A) NAFLD vs. non-NAFLD and MAFLD vs. non-MAFLD; (B) NAFLD-only vs. neither NAFLD nor MAFLD and MAFLD-only vs neither NAFLD nor MAFLD. The diamond indicates the pooled estimate. Red boxes are relative to the study size, and the black vertical lines indicate the 95% CIs around the effect size estimate. MAFLD: Metabolic-associated fatty liver disease; NAFLD: Non-alcoholic fatty liver disease.

The pooled results revealed that both NAFLD (n = 10,736) (HR = 1.18, 95% CI: 1.04–1.33; Q -statistic -*P *= 0.11, I^2^ = 55%) and MAFLD (n = 12,873) (HR = 1.30, 95% CI: 1.20–1.40, Q -statistic -*P* = 0.53, I^2^ = 0%) were associated with a statistically significant increased risk of all-cause death [29,34,36], with a nonsignificant subgroup difference (*P* = 0.19) (Fig 4). When data were classified according to the absence and/or presence of NAFLD and MAFLD, two studies were collected [26,33], which revealed that both the MAFLD-only group (n = 17,164), and the NAFLD-only group (n = 6,373) were not significantly associated with increased risk of all-cause death (MAFLD-only group: HR = 1.24, 95% CI: 0.73–2.12, Q -statistic -*P* < 0.01, I^2^ = 87%; NAFLD-only group: HR = 0.96, 95% CI: 0.72–1.29, Q -statistic -*P* = 0.89, I^2^ = 0%).

### Publication bias and sensitivity analysis

In this study, publication bias was not tested because of the limited number of included studies (N < 10) [37]. A sensitivity analysis with a fixed model yielded consistent results (S1 and S2 Figs in S1 File).

### Quality assessment

The GRADE assessment revealed low certainty for CVD (NAFLD only group vs. neither NAFLD nor MAFLD group), cardiovascular death (NAFLD only group vs. neither NAFLD nor MAFLD group), and all-cause mortality (MAFLD group vs non-MAFLD group), and very low certainty for other results (S6 Table in S1 File).

## Discussion

In this meta-analysis of eleven cohort studies encompassing 11,995,994 individuals, we made several critical discoveries regarding the cardiovascular risk associated with NAFLD and MAFLD: 1. Both NAFLD and MAFLD were significantly associated with an increased risk of CVD and all-cause mortality; 2. Subgroup analysis revealed that individuals in the MAFLD-only group had a numerically greater risk of cardiovascular mortality than those in the NAFLD-only group did, although this difference was not statistically significant.

A recent meta-analysis by Mantovani et al. [38] revealed that both NAFLD and MAFLD are associated with a statistically significant increase in the risk of cardiovascular events, whereas the association between a cardiovascular event and either NAFLD or MAFLD was not significantly different (Z score = 1.66, *P *= 0.097). In 2022, Zhou et al. [39] discussed the rationale for the definition of MAFLD and the clinical importance of MAFLD for cardiovascular-related diseases and concluded that patients with MAFLD are at greater risk of developing CVD than those with NAFLD; however, the exact mechanism of the association between MAFLD and CVD risk remains unknown. Our results are in line with Zho’s [39] findings. In addition, our data revealed borderline non-significant group differences between patients with NAFLD and MAFLD with associated CV events (*P* for interaction = 0.11). When cardiovascular events were further pooled for the MAFLD-only and NAFLD-only groups, the NAFLD-only group was associated with a statistically significant increase in cardiovascular risk, whereas the MAFLD-only group was not. Additionally, the CVD risk associated with either definition was not significantly different (*P* = 0.22) according to the comparison of HRs between the NAFLD-only group and the MAFLD-only group (Fig 2). Importantly, in the MAFLD-only group, although the two included studies showed a statistically significant increase in the CVD risk, the pooled result was not significant because of the high degree of heterogeneity (I^2^ = 88%). It cannot be ruled out that adding more studies might lead to significant differences between the MAFLD and NAFLD groups. Therefore, whether there is a difference in the risk of CV events between NAFLD and MAFLD needs to be further explored. Despite the extensive body of research on the roles of NAFLD and MAFLD in CVD risk, the results remain controversial.

With respect to cardiovascular mortality and all-cause mortality, in 2021, Nguyen et al. published a study that included 2997 subjects with NAFLD and/or MAFLD who were followed up for over 15 years; in that study, the MAFLD-only group had the highest cumulative all-cause mortality (26%), followed by the NAFLD + MAFLD group (fatty liver that met both the NAFLD and MAFLD criteria) (21.1%) and the NAFLD-only group (10.6), with a similar difference across the three groups in terms of CV-related mortality (*P* = 0.002) [40]. Similarly, a meta-analysis conducted by Virk et al. revealed that subjects diagnosed with MAFLD presented a significantly elevated risk of cardiovascular mortality compared to those diagnosed with NAFLD (risk ratio (RR)=1.48, 95% CI: 1.11–1.98). Furthermore, patients with MAFLD also had a greater risk of all-cause mortality (RR = 2.80, 95% CI: 2.39–3.28) [41]. While the present study and that of Virk et al. reached similar conclusions, there are notable differences. First, the statistical methodologies used differ; Virk et al. computed effect sizes as crude odds ratios, whereas we utilized adjusted HRs, resulting in differing levels of conclusion strengths. Additionally, we introduced subgroups such as NAFLD-only and MAFLD-only to underscore their specific effects.

Transitioning to the MAFLD nomenclature amplifies awareness of fatty liver disease, eschewing the requirement to rule out viral and alcoholic hepatitis, as opposed to the NAFLD classification. Moreover, it offers a more holistic view of metabolic dysfunction. The pivotal distinction between MAFLD and NAFLD lies in their diagnostic criteria. Through subgroup analysis, we validated the rationale behind the MAFLD nomenclature in this study. The majority of the articles included in this review were from Asia. Owing to the scarcity of articles at our disposal, we were unable to conduct a detailed subgroup analysis. Future research should explore possible geographical variances in the designation of NAFLD and MAFLD, potentially shedding light on regional nuances and contributing to a more refined understanding of fatty liver disease across different demographics.

The present study contributes to the ongoing debate by directly comparing the cardiovascular and mortality risks associated with NAFLD and MAFLD using a meta-analysis approach. The inconsistent results may stem from differences in diagnostic criteria for NAFLD and MAFLD, variations in the study populations (e.g., age, sex, comorbidities), and methodological differences. The heterogeneity of CVD outcomes across studies may also contribute to the conflicting findings. For example, we utilized a robust dataset comprising 11 cohort studies with a substantial sample size of 11,995,994 patients to examine the differential impacts of NAFLD and MAFLD on cardiovascular events, cardiovascular death, and all-cause mortality. GRADE assessment revealed low to very low certainty in the outcomes, partly due to heterogeneity, which reinforces the complexity of the issue and underscores the need for further research to clarify these discrepancies and better understand the relationship between these conditions and cardiovascular outcomes. To address these discrepancies, future research should aim to standardize diagnostic criteria for NAFLD and MAFLD to ensure consistency across studies. Large-scale, multicenter studies with diverse populations are needed to validate findings and enhance generalizability.

To the best of our knowledge, the present study stands as the most exhaustive one, uniquely summarizing outcomes for both cardiovascular events and all-cause mortality. A pivotal focus was the understudied population segment that met only one set of diagnostic criteria, highlighting a subset that, regardless of terminology, would be overlooked by one diagnosis or the other. Our findings highlight that the MAFLD-only group is more strongly correlated with cardiovascular death than the NAFLD-only group is, lending credence to the argument for rebranding NAFLD to MAFLD.

The limitations of our study include its foundation on observational design, which inherently limits causal inference. Moreover, the heterogeneity in the diagnostic criteria for hepatic steatosis across the included studies introduced potential methodological variability. The diagnostic approaches used varied and included ultrasound, the fatty liver index, and K-NAFLD methods, which may have influenced the consistency of disease classification and contributed to heterogeneity. In addition, the limited number of available studies precluded comprehensive meta-regression analyses, limiting our ability to fully explore sources of heterogeneity. Subgroup analyses were particularly challenging due to the restricted dataset, resulting in inconclusive differences that warrant further investigation. In addition, a new term MASLD has been recently proposed with a different set of criteria than that for the NAFLD definition [42,43]. Our study did not include comparisons of MASLD with the other two types. This change in terminology may stimulate further meta-analyses comparing MAFLD, MASLD, and NAFLD. Subsequent studies are needed to validate our conclusions further.

## Conclusion

Our findings indicate that both MAFLD and NAFLD are significantly associated with an increased risk of cardiovascular events and all-cause mortality. Notably, individuals diagnosed with MAFLD have a greater incidence of cardiovascular death than those with NAFLD. These findings suggest that the criteria for MAFLD may be instrumental in identifying patients at elevated risk for cardiovascular mortality, thereby facilitating targeted interventions and monitoring strategies. However, our results need to be interpreted with caution, and more prospective studies are needed to confirm our findings because of the limited number of available studies.

## Supporting information

S1 File S1 Fig. Forest plot analysis of NAFLD/MAFLD and cardiovascular mortality risk. S2 Fig. Forest plot analysis of NAFLD/MAFLD and all-cause mortality risk. S1 Table. PRISMA checklist. S2 Table. Excluded studies. S3 Table. Newcastle-Ottawa Scale evaluation of included observational studies. S4 Table. Diagnostic criteria for NAFLD and MAFLD in included studies. S5 Table. Search strategy. S6 Table. GRADE assessment.
(DOCX)

## Abbreviations

CIs: confidence intervals
RR: relative risk
HR: hazard ratio
OR: odds ratio
NOS: Newcastle‒Ottawa Scale
NAFLD: nonalcoholic fatty liver disease
MAFLD: metabolic dysfunction-associated fatty liver disease
CVD: cardiovascular disease
