# Supplementary material for: Comparative association between NAFLD and MAFLD with cardiovascular events and mortality: Evidence from observational studies
Source: PLoS One. 2025 Jun 13;20(6):e0312650. doi: 10.1371/journal.pone.0312650 (PMC12165386; doi:10.1371/journal.pone.0312650)

# Supplementary Content

Comparative Association between NAFLD and MAFLD with Cardiovascular Events, and Mortality: A Meta-Analysis

**S1 Table. PRISMA Checklist.**

**S2 Table. Excluded Studies.**

**S3 Table. Newcastle-Ottawa Scale Evaluation of Included Observational Studies.**

**S4 Table. Diagnostic Criteria for NAFLD and MAFLD in Included Studies.**

**S5 Table. Search strategy.**

**S6 Table. GRADE assessment.**

**S1 Fig. Forest Plot Analysis of NAFLD/MAFLD and Cardiovascular Mortality Risk.**

**S2 Fig. Forest Plot Analysis of NAFLD/MAFLD and All-Cause Mortality Risk.**

This supplementary material has been provided by the authors to give readers additional information about their work.

**S1 Table. PRISMA Checklist**

| **Section and Topic** | **Item #** | **Checklist item** | **Location where item is reported** |
| --- | --- | --- | --- |
| **TITLE** | | |  |
| Title | 1 | Identify the report as a systematic review. | 1 |
| **ABSTRACT** | | |  |
| Abstract | 2 | See the PRISMA 2020 for Abstracts checklist. | 2-3 |
| **INTRODUCTION** | | |  |
| Rationale | 3 | Describe the rationale for the review in the context of existing knowledge. | 5 |
| Objectives | 4 | Provide an explicit statement of the objective(s) or question(s) the review addresses. | 5 |
| **METHODS** | | |  |
| Eligibility criteria | 5 | Specify the inclusion and exclusion criteria for the review and how studies were grouped for the syntheses. | 6 |
| Information sources | 6 | Specify all databases, registers, websites, organisations, reference lists and other sources searched or consulted to identify studies. Specify the date when each source was last searched or consulted. | 5-6 |
| Search strategy | 7 | Present the full search strategies for all databases, registers and websites, including any filters and limits used. | 6 |
| Selection process | 8 | Specify the methods used to decide whether a study met the inclusion criteria of the review, including how many reviewers screened each record and each report retrieved, whether they worked independently, and if applicable, details of automation tools used in the process. | 6 |
| Data collection process | 9 | Specify the methods used to collect data from reports, including how many reviewers collected data from each report, whether they worked independently, any processes for obtaining or confirming data from study investigators, and if applicable, details of automation tools used in the process. | 6 |
| Data items | 10a | List and define all outcomes for which data were sought. Specify whether all results that were compatible with each outcome domain in each study were sought (e.g. for all measures, time points, analyses), and if not, the methods used to decide which results to collect. | 7 |
|  | 10b | List and define all other variables for which data were sought (e.g. participant and intervention characteristics, funding sources). Describe any assumptions made about any missing or unclear information. | 7 |
| Study risk of bias assessment | 11 | Specify the methods used to assess risk of bias in the included studies, including details of the tool(s) used, how many reviewers assessed each study and whether they worked independently, and if applicable, details of automation tools used in the process. | 7 |
| Effect measures | 12 | Specify for each outcome the effect measure(s) (e.g. risk ratio, mean difference) used in the synthesis or presentation of results. | 7 |
| Synthesis methods | 13a | Describe the processes used to decide which studies were eligible for each synthesis (e.g. tabulating the study intervention characteristics and comparing against the planned groups for each synthesis (item #5)). | 7 |
|  | 13b | Describe any methods required to prepare the data for presentation or synthesis, such as handling of missing summary statistics, or data conversions. | 7 |
|  | 13c | Describe any methods used to tabulate or visually display results of individual studies and syntheses. | 7 |
|  | 13d | Describe any methods used to synthesize results and provide a rationale for the choice(s). If meta-analysis was performed, describe the model(s), method(s) to identify the presence and extent of statistical heterogeneity, and software package(s) used. | 7 |
|  | 13e | Describe any methods used to explore possible causes of heterogeneity among study results (e.g. subgroup analysis, meta-regression). | 7 |
|  | 13f | Describe any sensitivity analyses conducted to assess robustness of the synthesized results. | 7 |
| Reporting bias assessment | 14 | Describe any methods used to assess risk of bias due to missing results in a synthesis (arising from reporting biases). | 7 |
| Certainty assessment | 15 | Describe any methods used to assess certainty (or confidence) in the body of evidence for an outcome. | 7-8 |
| **RESULTS** | | |  |
| Study selection | 16a | Describe the results of the search and selection process, from the number of records identified in the search to the number of studies included in the review, ideally using a flow diagram. | 8 |
|  | 16b | Cite studies that might appear to meet the inclusion criteria, but which were excluded, and explain why they were excluded. | 8 |
| Study characteristics | 17 | Cite each included study and present its characteristics. | 8 |
| Risk of bias in studies | 18 | Present assessments of risk of bias for each included study. | 8 |
| Results of individual studies | 19 | For all outcomes, present, for each study: (a) summary statistics for each group (where appropriate) and (b) an effect estimate and its precision (e.g. confidence/credible interval), ideally using structured tables or plots. | 9-10 |
| Results of syntheses | 20a | For each synthesis, briefly summarise the characteristics and risk of bias among contributing studies. | 9-10 |
|  | 20b | Present results of all statistical syntheses conducted. If meta-analysis was done, present for each the summary estimate and its precision (e.g. confidence/credible interval) and measures of statistical heterogeneity. If comparing groups, describe the direction of the effect. | 9-10 |
|  | 20c | Present results of all investigations of possible causes of heterogeneity among study results. | 9-10 |
|  | 20d | Present results of all sensitivity analyses conducted to assess the robustness of the synthesized results. | 10 |
| Reporting biases | 21 | Present assessments of risk of bias due to missing results (arising from reporting biases) for each synthesis assessed. | 10 |
| Certainty of evidence | 22 | Present assessments of certainty (or confidence) in the body of evidence for each outcome assessed. | 11 |
| **DISCUSSION** | | |  |
| Discussion | 23a | Provide a general interpretation of the results in the context of other evidence. | 11 |
|  | 23b | Discuss any limitations of the evidence included in the review. | 14 |
|  | 23c | Discuss any limitations of the review processes used. | 14 |
|  | 23d | Discuss implications of the results for practice, policy, and future research. | 13-14 |
| **OTHER INFORMATION** | | |  |
| Registration and protocol | 24a | Provide registration information for the review, including register name and registration number, or state that the review was not registered. | 5-6 |
|  | 24b | Indicate where the review protocol can be accessed, or state that a protocol was not prepared. | 5-6 |
|  | 24c | Describe and explain any amendments to information provided at registration or in the protocol. | 5-6 |
| Support | 25 | Describe sources of financial or non-financial support for the review, and the role of the funders or sponsors in the review. | 15 |
| Competing interests | 26 | Declare any competing interests of review authors. | 15 |
| Availability of data, code and other materials | 27 | Report which of the following are publicly available and where they can be found: template data collection forms; data extracted from included studies; data used for all analyses; analytic code; any other materials used in the review. | 16 |

*From:*  Page MJ, McKenzie JE, Bossuyt PM, Boutron I, Hoffmann TC, Mulrow CD, et al. The PRISMA 2020 statement: an updated guideline for reporting systematic reviews. BMJ 2021;372:n71. doi: 10.1136/bmj.n71

**S2 Table. Excluded Studies**

| **Studies excluded** | **Reasons** |
| --- | --- |
| Alharthi, 2022[1] | This is a review. |
| Chen. 2021[2] | Not target contrast: MAFLD subtypes. |
| Cheng, 2024[3] | Not target contrast: NAFLD only vs both NAFLD and MAFLD; MAFLD only vs both NAFLD and MAFLD |
| Choe, 2024[4] | Not target contrast: MASLD vs MetALD vs ALD |
| Guerreiro, 2021[5] | Not target contrast: NAFLD vs MAFLD. |
| Huang, 2021[6] | Same population as Kim, 2021[7], but the data is less rigorous than Kim, 2021. |
| Kang, 2021[8] | This is a review. |
| Kim, 2022[9] | Not the target outcome: coronary risk surrogates, coronary artery disease, intermediate to high ASCVD risk. |
| Liu, 2021[10] | Not target contrast: MAFLD vs non-MAFLD; MAFLD overlapped with NAFLD vs MAFLD-only. |
| Ng, 2022[11] | This is a review. |
| Nguyen, 2021[12] | Not target contrast: NAFLD only vs both NAFLD and MAFLD vs MAFLD only. |
| Scheinberg, 2022[13] | This is a editotial. |
| Semmler, 2021[14] | No adjusted hazard ratio values. |
| Song, 2024[15] | Same population as Kim, 2024[16]. |
| Tamaki, 2021[17] | This is a letter. |
| Tsutsumi, 2021[18] | Not target contrast: NAFLD only vs both NAFLD and MAFLD vs MAFLD only. |
| Wang, 2022[19] | No adjusted hazard ratio values. |
| Younossi, 2022[20] | Not the target outcome: risk factors for all-cause and cause-specific mortality. |
| Zhang, 2021[21] | This is a cross-sectional study. |

1. Alharthi, J., et al., *Metabolic dysfunction-associated fatty liver disease: a year in review.* Curr Opin Gastroenterol, 2022. **38**(3): p. 251-260.

2. Chen, X., et al., *Are the different MAFLD subtypes based on the inclusion criteria correlated with all-cause mortality?* J Hepatol, 2021. **75**(4): p. 987-989.

3. Cheng, W.C., et al., *Comparison of all-cause mortality associated with non-alcoholic fatty liver disease and metabolic dysfunction-associated fatty liver disease in Taiwan MJ cohort.* Epidemiol Health, 2024. **46**: p. e2024024.

4. Choe, H.J., et al., *Steatotic liver disease predicts cardiovascular disease and advanced liver fibrosis: A community-dwelling cohort study with 20-year follow-up.* Metabolism, 2024. **153**: p. 155800.

5. Guerreiro, G.T.S., et al., *Does the risk of cardiovascular events differ between biopsy-proven NAFLD and MAFLD?* Hepatology International, 2021. **15**(2): p. 380-391.

6. Huang, Q., et al., *NAFLD or MAFLD: Which Has Closer Association With All-Cause and Cause-Specific Mortality?—Results From NHANES III.* Frontiers in Medicine, 2021. **8**.

7. Kim, D., et al., *Metabolic dysfunction-associated fatty liver disease is associated with increased all-cause mortality in the United States.* Journal of Hepatology, 2021. **75**(6): p. 1284-1291.

8. Kang, S.H., et al., *From nonalcoholic fatty liver disease to metabolic-associated fatty liver disease: Big wave or ripple?* Clinical and Molecular Hepatology, 2021. **27**(2): p. 257-269.

9. Kim, H., et al., *MAFLD Predicts the Risk of Cardiovascular Disease Better than NAFLD in Asymptomatic Subjects with Health Check-Ups.* Digestive Diseases and Sciences, 2022.

10. Liu, H.H., et al., *Metabolic-associated fatty liver disease and major adverse cardiac events in patients with chronic coronary syndrome: a matched case-control study.* Hepatol Int, 2021. **15**(6): p. 1337-1346.

11. Ng, C.H., D.Q. Huang, and M.H. Nguyen, *NAFLD versus MAFLD: Prevalence, Outcomes and Implications of a Change in Name.* Clinical and molecular hepatology, 2022.

12. Nguyen, V.H., et al., *Differential Clinical Characteristics and Mortality Outcomes in Persons With NAFLD and/or MAFLD.* Clinical Gastroenterology and Hepatology, 2021. **19**(10): p. 2172-2181.e6.

13. Scheinberg, A.R. and B.V. John, *MAFLD Versus NAFLD: Which Better Predicts the Risk of Atherosclerotic Cardiovascular Disease?* Digestive Diseases and Sciences, 2022.

14. Semmler, G., et al., *Metabolic Dysfunction-Associated Fatty Liver Disease (MAFLD)-Rather a Bystander Than a Driver of Mortality.* J Clin Endocrinol Metab, 2021. **106**(9): p. 2670-2677.

15. Song, R., et al., *Comparison of NAFLD, MAFLD and MASLD characteristics and mortality outcomes in United States adults.* Liver Int, 2024. **44**(4): p. 1051-1060.

16. Kim, D., et al., *Steatotic liver disease-associated all-cause/cause-specific mortality in the United States.* Aliment Pharmacol Ther, 2024.

17. Tamaki, N., et al., *Atherosclerotic cardiovascular disease risk difference in metabolic dysfunction-associated fatty liver disease and nonalcoholic fatty liver disease.* Hepatology Research, 2021. **51**(11): p. 1172-1173.

18. Tsutsumi, T., et al., *MAFLD better predicts the progression of atherosclerotic cardiovascular risk than NAFLD: Generalized estimating equation approach.* Hepatology Research, 2021. **51**(11): p. 1115-1128.

19. Wang, X., et al., *Metabolic Dysfunction-associated Fatty Liver Disease and Mortality Among Chinese Adults: a Prospective Cohort Study.* J Clin Endocrinol Metab, 2022. **107**(2): p. e745-e755.

20. Younossi, Z.M., et al., *Are there outcome differences between NAFLD and metabolic-associated fatty liver disease?* Hepatology, 2022.

21. Zhang, H.J., et al., *Cardiovascular and renal burdens of metabolic associated fatty liver disease from serial US national surveys, 1999-2016.* Chinese Medical Journal, 2021. **134**(13): p. 1593-1601.

**S3 Table. Newcastle-Ottawa Scale Evaluation of Included Observational Studies**

| Author  (Publication Year) | Newcastle-Ottawa Scale | | | | | | | | | |
| --- | --- | --- | --- | --- | --- | --- | --- | --- | --- | --- |
|  | Selection | | | Comparability | | | Outcome | | | Total |
|  | a | b | c | d | e | f | g | h | i |  |
| Han, 2024 | 1 | 1 | 1 | 1 | 1 | 1 | 1 | 1 | 0 | 8 |
| Jeong, 2021 | 1 | 1 | 1 | 1 | 1 | 1 | 1 | 1 | 0 | 8 |
| Kim, 2021 | 1 | 1 | 1 | 1 | 1 | 1 | 1 | 1 | 0 | 8 |
| Kim, 2023 | 1 | 1 | 1 | 1 | 1 | 1 | 1 | 1 | 0 | 8 |
| Kim, 2024 | 1 | 1 | 1 | 1 | 1 | 1 | 1 | 1 | 0 | 8 |
| Lee, 2021 | 1 | 1 | 1 | 1 | 1 | 1 | 1 | 1 | 0 | 8 |
| liang, 2022 | 1 | 1 | 1 | 1 | 1 | 1 | 0 | 1 | 1 | 8 |
| moon, 2021 | 1 | 1 | 1 | 1 | 1 | 1 | 0 | 1 | 0 | 7 |
| Niriella, 2021 | 1 | 1 | 1 | 1 | 1 | 1 | 1 | 1 | 1 | 9 |
| Yoneda, 2021 | 1 | 1 | 1 | 1 | 1 | 1 | 1 | 1 | 0 | 8 |
| Yoo, 2023 | 1 | 1 | 1 | 1 | 1 | 1 | 1 | 1 | 0 | 8 |

1. Representativeness of the exposed cohort.
2. Selection of the non-exposed cohort.
3. Ascertainment of exposure.
4. Demonstration that outcome of interest was not present at start of study.
5. Comparability of cohorts on the basis of the design or analysis (adjusted for age).
6. Comparability of cohorts on the basis of the design or analysis (adjusted for any other factor).
7. Assessment of outcome.
8. Was follow-up long enough for outcomes to occur. (At least 1 year to eliminate seasonal effects of influenza).
9. Adequacy of follow-up of cohorts.

**S4 Table. Diagnostic Criteria for NAFLD and MAFLD in Included Studies**

| References (first author, year, country/region) | NAFLD | MAFLD |
| --- | --- | --- |
| Han, 2024, Korea | Presence of hepatic steatosis (K-NAFLD score > 0.884) without alcohol consumption and chronic viral hepatitis B and C, which were defined using the International Classification of Diseases 10th Revision (ICD-10) code of B18 | Presence of hepatic steatosis (K-NAFLD score > 0.884) with 1 or more of the following:  (1) BMI ≥23 kg/m2;  (2) fasting blood sugar ≥126 mg/dL and/or prescription of antidiabetic drugs;  (3) ≥2 metabolic risk abnormalities:  a. WC ≥90 cm for men and ≥80 cm for women;  b. BP ≥130/85 mm Hg or specific drug treatment; c. TG ≥150 mg/dL or specific drug treatment;  d. HDL-C <40 mg/dL for men and <50 mg/dL for women;  e. prediabetes (fasting blood sugar between 100 to 125 mg/dL) |
| Jeong, 2021, South Korean | Presence of hepatic steatosis (K-NAFLD score > 0.884) without alcohol consumption and chronic viral hepatitis B and C, which were defined using the International Classification of Diseases 10th Revision (ICD-10) code of B18 | Presence of hepatic steatosis (K-NAFLD score > 0.884) with 1 or more of the following:  (1) BMI ≥23 kg/m2;  (2) fasting blood sugar ≥126 mg/dL and/or prescription of antidiabetic drugs;  (3) ≥2 metabolic risk abnormalities:  a. WC ≥90 cm for men and ≥80 cm for women;  b. BP ≥130/85 mm Hg or specific drug treatment; c. TG ≥150 mg/dL or specific drug treatment;  d. HDL-C <40 mg/dL for men and <50 mg/dL for women;  e. prediabetes (fasting blood sugar between 100 to 125 mg/dL) |
| Kim, 2021, North America | Presence of hepatic steatosis (ultrasound evidence of fatty liver), without significant alcohol consumption (> 21 drinks/week in men and > 14 drinks/week in women) and/or viral hepatitis (positive serum hepatitis C antibody and/or positive serum hepatitis B surface antigen) | Presence of hepatic steatosis (ultrasound evidence of fatty liver) with 1 or more of the following:  (1) BMI ≥ 25 kg/m2.  (2) diabetes mellitus  (3) ≥2 metabolic risk abnormalities:  a. WC ≥ 102 cm for men and 88 ≥ cm for women;  b. BP ≥ 130/85 mmHg or specific drug treatment;  c. fasting plasma TG ≥ 150 mg/dl or specific drug treatment;  d. plasma HDL-C < 40 mg/dl for men and < 50 mg/dl for women or specific drug treatment,  e. prediabetes (fasting glucose 100-125 mg/dl or hemoglobin A1c 5.7%-6.4%,  f. HOMA-IR ≥ 2.5,  g. plasma hs-CRP > 2 mg/L |
| Kim, 2023, South Korea | Presence of hepatic steatosis (ultrasound evidence of fatty liver), without significant alcohol consumption (> 21 drinks/week in men and > 14 drinks/week in women) and/or viral hepatitis (positive serum hepatitis C antibody and/or positive serum hepatitis B surface antigen). | Presence of hepatic steatosis (Fatty Liver Index ≥ 30) with 1 or more of the following:  (1) BMI ≥23 kg/m2;  (2) diabetes mellitus  (3) ≥2 metabolic risk abnormalities:  a. WC ≥90 cm in men and ≥80 cm women;  b. BP ≥130/85 mmHg or specific drug treatmentc.  c. TG ≥150 mg/dL or specific drug treatment;  d. HDL-C <40 mg/dL in men and <50 mg/dL in women or specific drug treatment;  e. fasting glucose ≥100 mg/dL;  f. HOMA-IR ≥2.5;  g. hs-CRP >2 mg/L |
| Kim, 2024, America | Presence of hepatic steatosis (ultrasound evidence of fatty liver), without significant alcohol consumption (> 21 drinks/week in men and > 14 drinks/week in women) and/or viral hepatitis (positive serum hepatitis C antibody and/or positive serum hepatitis B surface antigen). | Presence of hepatic steatosis (Fatty Liver Index ≥ 30) with 1 or more of the following:  (1) BMI ≥23 kg/m2;  (2) diabetes mellitus  (3) ≥2 metabolic risk abnormalities:  a. WC ≥90 cm in men and ≥80 cm women;  b. BP ≥130/85 mmHg or specific drug treatmentc.  c. TG ≥150 mg/dL or specific drug treatment;  d. HDL-C <40 mg/dL in men and <50 mg/dL in women or specific drug treatment;  e. fasting glucose ≥100 mg/dL;  f. HOMA-IR ≥2.5;  g. hs-CRP >2 mg/L |
| Lee, 2021, Korea | Presence of hepatic steatosis (Fatty Liver Index ≥ 30) without excessive alcohol consumption (30 g/d in men and 20 g/d in women; assuming 1 drink = 10 g ethanol) or concomitant liver disease. | Presence of hepatic steatosis (Fatty Liver Index ≥ 30) with 1 or more of the following:  (1) BMI ≥23 kg/m2;  (2) diabetes mellitus  (3) ≥2 metabolic risk abnormalities:  a. WC ≥90 cm in men and ≥80 cm women;  b. BP ≥130/85 mmHg or specific drug treatmentc.  c. TG ≥150 mg/dL or specific drug treatment;  d. HDL-C <40 mg/dL in men and <50 mg/dL in women or specific drug treatment;  e. fasting glucose ≥100 mg/dL;  f. HOMA-IR ≥2.5;  g. hs-CRP >2 mg/L |
| liang, 2022, China | Presence of hepatic steatosis (ultrasound evidence of fatty liver), in the absence of excessive alcohol consumption and other concomitant liver diseases (viral hepatitis, hepatolenticular degeneration, drug-induced hepatitis, autoimmune hepatitis, etc) | Presence of hepatic steatosis (ultrasound evidence of fatty liver) with 1 or more of the following:  (1) BMI ≥ 23.0 in Asia;  (2) diabetes;  (3) ≥2 metabolic risk abnormalities:  a. WC ≥ 90 cm in Asian men and ≥ 80 cm in Asian women;  b. BP ≥ 130/85 mmHg or specific drug treatment;  c. TG ≥ 1.70 mmol/L or specific drug treatment;  d. HDL-C < 1.0 mmol/L/1.3 mmol/L for men/women or specific drug treatment;  e. prediabetes (fasting glucose = 5.6-6.9 mmol/L and/or hemoglobin A1c = 5.7%-6.4% in participants without a prior diabetes diagnosis);  f. HOMA-IR ≥ 2.5;  g. hs-CRP level > 2 mg/L. |
| Moon, 2021, South Korean | Presence of hepatic steatosis (Fatty Liver Index ≥ 60) without other etiologies of chronic liver disease. | Presence of hepatic steatosis (Fatty Liver Index ≥ 60) with 1 or more of the following:  (1) BMI ≥ 23.0 in Asia;  (2) type 2 diabetes mellitus;  (3) ≥2 metabolic risk abnormalities:  a. WC ≥102/88 cm in Caucasian men and women or ≥90/80 cm in Asian men and women  b. BP ≥130/85 mmHg or specific drug treatment  c. plasma TG ≥150 mg/dl (≥1.70 mmol/L) or specific drug treatment;  d. plasma HDL-C <40 mg/dl (<1.0 mmol/L) for men and <50 mg/dl (<1.3 mmol/L) for women or specific drug treatment.  e. prediabetes (i.e., fasting glucose levels 100 to 125 mg/dl [5.6 to 6.9 mmol/L], or 2-hour post-load glucose levels 140 to 199 mg/dl [7.8 to 11.0 mmol] or Hemoglobin A1c 5.7% to 6.4% [39 to 47 mmol/mol])  f. HOMA-IR ≥ 2.5  g. Plasma hs-CRP >2 mg/L |
| Niriella，2021, Sri Lanka | Presence of hepatic steatosis (ultrasound evidence of fatty liver), safe alcohol consumption (Asian standards: <14 units/week for men, <7 units/week for females) and absence of Hepatitis B and C markers. | Presence of hepatic steatosis (ultrasound evidence of fatty liver) with 1 or more of the following:  (1) BMI ≥ 23.0 in Asia;  (2) diabetes (fasting blood sugar >125 mg/dL or Hemoglobin A1c >6.4% or on treatment);  (3) ≥2 metabolic risk abnormalities:  a. WC ≥ 90cm for men, WC ≥ 80cm for women;  b. BP ≥ 130/85 mmHg or on treatment;  c. TG >150mg/dL or on treatment;  d. HDL-C < 40mg/dL for men and <50 mg/dL for women or on treatment;  e. fasting blood sugar between 100–125 mg/dL or Hemoglobin A1c 5.7–6.4%. |
| Yoneda, 2021, Japan | Presence of hepatic steatosis (Fatty Liver Index ≥ 60) in the absence of excessive alcohol consumption and other concomitant liver diseases. | Presence of hepatic steatosis (Fatty Liver Index ≥ 60) with 1 or more of the following:  (1) BMI ≥ 23.0 in Asia;  (2) diabetes  (3) ≥2 metabolic risk abnormalities:  a. WC ≥ 102 in men and 88 cm in women;  b. BP ≥ 130/85 mmHg or specific drug treatment,  c. TG ≥ 150 mg/dl or specific drug treatment;  d HDL-C ＜ 40 mg/dl for men and＜ 50mg/dl for women or specific drug treatment,  e. prediabetes (i.e., fasting glucose levels 100–125 mg/dl, or 2-h post load glucose levels 140 to 199 mg/dl or Hemoglobin A1c 5.7–6.4%,  f. HOMA-IR ≥ 2.5  g. Plasma hs-CRP ＞ 2 mg/L.  Since the study database does not have data on 2-h postload glucose levels, HOMA-R, and hs-CRP, these factors were not used. |
| Yoo, 2023, Korea | Presence of hepatic steatosis (ultrasound evidence of fatty liver), without significant alcohol consumption (> 21 drinks/week in men and > 14 drinks/week in women) and/or viral hepatitis (positive serum hepatitis C antibody and/or positive serum hepatitis B surface antigen). | Presence of hepatic steatosis (Fatty Liver Index ≥ 30) with 1 or more of the following:  (1) BMI ≥23 kg/m2;  (2) diabetes mellitus  (3) ≥2 metabolic risk abnormalities:  a. WC ≥90 cm in men and ≥80 cm women;  b. BP ≥130/85 mmHg or specific drug treatmentc.  c. TG ≥150 mg/dL or specific drug treatment;  d. HDL-C <40 mg/dL in men and <50 mg/dL in women or specific drug treatment;  e. fasting glucose ≥100 mg/dL;  f. HOMA-IR ≥2.5;  g. hs-CRP >2 mg/L |

Abbreviations: BMI: body mass index; WC: waist circumference; BP: blood pressure; TG: triglycerides; HDL-C: high-density lipoprotein-cholesterol; hs-CRP: high-sensitivity Creactive protein; HOMA-IR: homeostasis model assessment of insulin resistance score

Note: The method of fatty liver assessment in each article was consistent for NAFLD and MAFLD.

**S5 Table. Search strategy**

| Pubmed (n = 425) |
| --- |
| (((metabolic dysfunction-associated fatty liver disease) OR (MAFLD)) AND ((Non-alcoholic fatty liver disease) OR (NAFLD))) AND (((cardiovascular) OR (death)) OR (mortality)) |
| Embase (n = 422) |
| #1 mafld  #2 metabolic dysfunction-associated fatty liver disease  #3 #1 OR #2  #4 nafld  #5 Non-alcoholic fatty liver disease  #6 #3 OR #4  #7 #3AND #6  #8 cardiovascular  #9 death  #10 mortality  #11 #8 OR #9 OR #10  #12 #7 AND #11 |
| Cochrane (n = 10) |
| #1 mafld  #2 metabolic dysfunction-associated fatty liver disease  #3 #1 OR #2  #4 nafld  #5 Non-alcoholic fatty liver disease  #6 #3 OR #4  #7 #3AND #6  #8 cardiovascular  #9 death  #10 mortality  #11 #8 OR #9 OR #10  #12 #7 AND #11 |

**S6 Table. GRADE assessment.**

| **Quality assessment** | | | | | | | **No of patients** | | **Effect** | | **Quality** | **Importance** |  |
| --- | --- | --- | --- | --- | --- | --- | --- | --- | --- | --- | --- | --- | --- |
|  |  |  |  |  |  |  |  |  |  |  |  |  |  |
| **No of studies** | **Design** | **Risk of bias** | **Inconsistency** | **Indirectness** | **Imprecision** | **Other considerations** | **CVD** | **Control** | **Relative (95% CI)** | **Absolute** |  |  |  |
| **cardiovascular disease - NAFLD vs non-NAFLD** | | | | | | | | | | | | |  |
| 6 | observational studies | no serious risk of bias | very serious^1^ | no serious indirectness | no serious imprecision | none | - | - | HR 1.30 (1.13, 1.49) | - | ÅOOO VERY LOW | IMPORTANT |  |
|  |  |  |  |  |  |  |  | - |  | - |  |  |  |
| **cardiovascular disease - MAFLD vs non-MAFLD** | | | | | | | | | | | | |  |
| 6 | observational studies | no serious risk of bias | very serious^1^ | no serious indirectness | no serious imprecision | none | - | - | HR 1.54 (1.32, 1.81) | - | ÅOOO VERY LOW | IMPORTANT |  |
|  |  |  |  |  |  |  |  | - |  | - |  |  |  |
| **cardiovascular disease - NAFLD only vs neither NAFLD nor MAFLD** | | | | | | | | | | | | |  |
| 2 | observational studies | no serious risk of bias | no serious inconsistency | no serious indirectness | no serious imprecision | none | - | - | HR 1.09 (1.03, 1.15) | - | ÅÅOO LOW | IMPORTANT |  |
|  |  |  |  |  |  |  |  | - |  | - |  |  |  |
| **cardiovascular disease - MAFLD only vs neither NAFLD nor MAFLD** | | | | | | | | | | | | |  |
| 2 | observational studies | no serious risk of bias | very serious^1^ | no serious indirectness | serious^3^ | none | - | - | HR 2.91 (0.60, 14.04) | - | ÅOOO VERY LOW | IMPORTANT |  |
|  |  |  |  |  |  |  |  | - |  | - |  |  |  |
| **cardiovascular death - NAFLD vs non-NAFLD** | | | | | | | | | | | | |  |
| 2 | observational studies | no serious risk of bias | very serious^1^ | no serious indirectness | no serious imprecision | none | - | - | HR 0.96 (0.77, 1.20) | - | ÅOOO VERY LOW | CRITICAL |  |
|  |  |  |  |  |  |  |  | -- |  | - |  |  |  |
| **cardiovascular death - MAFLD vs non-MAFLD** | | | | | | | | | | | | |  |
| 2 | observational studies | no serious risk of bias | Serious^2^ | no serious indirectness | no serious imprecision | none | - | - | HR 1.05 (0.88, 1.14) | - | ÅOOO VERY LOW | CRITICAL |  |
|  |  |  |  |  |  |  |  | - |  | - |  |  |  |
| **cardiovascular death - NAFLD only vs neither NAFLD nor MAFLD** | | | | | | | | | | | | |  |
| 3 | observational studies | no serious risk of bias | no serious inconsistency | no serious indirectness | no serious imprecision | none | - | - | HR 1.10 (0.95, 1.28) | - | ÅÅOO LOW | CRITICAL |  |
|  |  |  |  |  |  |  |  | - |  | - |  |  |  |
| **cardiovascular death - MAFLD only vs neither NAFLD nor MAFLD** | | | | | | | | | | | | |  |
| 3 | observational studies | no serious risk of bias | no serious inconsistency | no serious indirectness | serious^3^ | none | - | - | HR 1.46 (1.40, 1.51) | - | ÅOOO VERY LOW | CRITICAL |  |
|  |  |  |  |  |  |  |  | - |  | - |  |  |  |
| **all-cause - NAFLD vs non-NAFLD** | | | | | | | | | | | | |  |
| 3 | observational studies | no serious risk of bias | Serious^2^ | no serious indirectness | no serious imprecision | none | - | - | HR 1.18 (1.04, 1.33) | - | ÅOOO VERY LOW | CRITICAL |  |
|  |  |  |  |  |  |  |  | - |  | - |  |  |  |
| **all-cause - MAFLD vs non-MAFLD** | | | | | | | | | | | | |  |
| 3 | observational studies | no serious risk of bias | no serious inconsistency | no serious indirectness | no serious imprecision | none | - | - | HR 1.30 (1.20, 1.40) | - | ÅÅOO LOW | CRITICAL |  |
|  |  |  |  |  |  |  |  | - |  | - |  |  |  |
| **all cause death - NAFLD only vs neither NAFLD nor MAFLD** | | | | | | | | | | | | |  |
| 2 | observational studies | no serious risk of bias | no serious inconsistency | no serious indirectness | serious^3^ | none | - | - | HR 0.96 (0.72, 1.29) | - | ÅOOO VERY LOW | CRITICAL |  |
|  |  |  |  |  |  |  |  | - |  | - |  |  |  |
| **all cause death - MAFLD only vs neither NAFLD nor MAFLD** | | | | | | | | | | | | |  |
| 2 | observational studies | no serious risk of bias | very serious^1^ | no serious indirectness | serious^3^ | none | - | - | HR 1.24 (0.73, 2.12) | - | ÅOOO VERY LOW | CRITICAL |  |
|  |  |  |  |  |  |  |  | - |  | - |  |  |  |

^1^ I^2^ > 75%
^2^ 50% < I^2^ ≤ 75%

^3^ The 95% CI include 1.00.

**S1 Fig. Forest Plot Analysis of NAFLD/MAFLD and Cardiovascular Mortality Risk**

Forest plot for the association between NAFLD/MAFLD and CV death

(A)

**
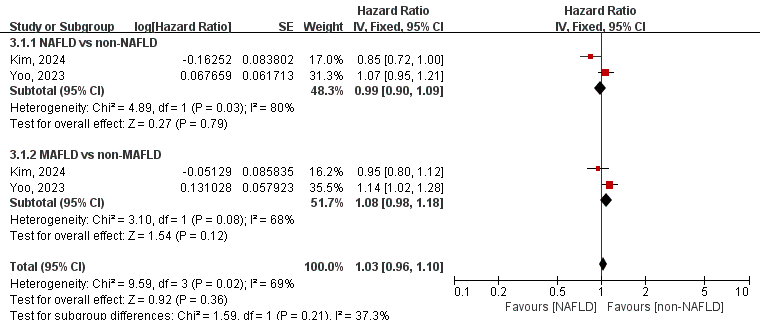
**

(B)

**
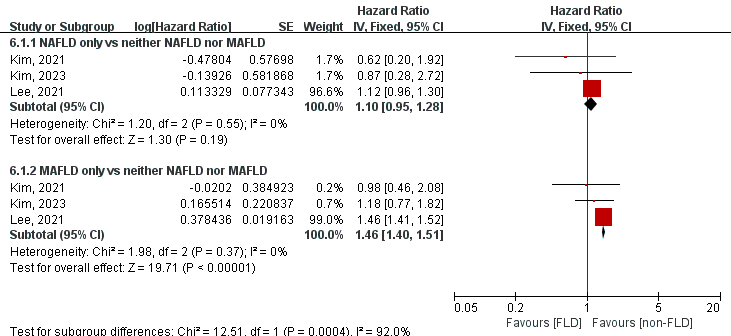
**

**S2 Fig. Forest Plot Analysis of NAFLD/MAFLD and All-Cause Mortality Risk**

Forest plot for the association between NAFLD/MAFLD and all-cause

(A)


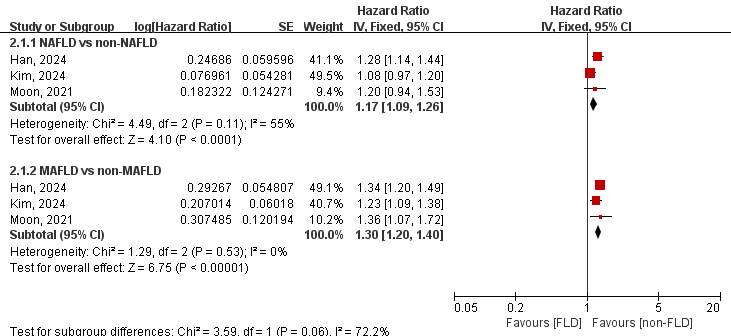


(B)


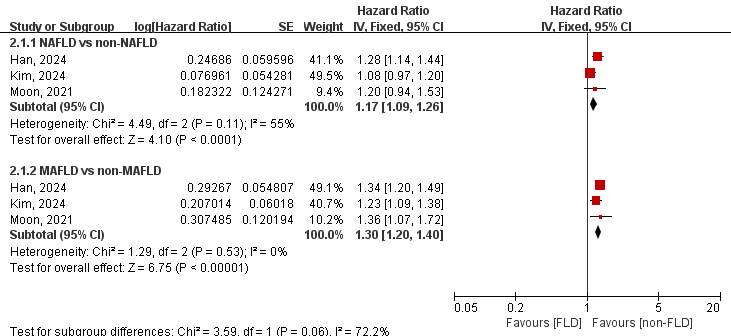

Supplement: S1 File — S1 Fig. Forest plot analysis of NAFLD/MAFLD and cardiovascular mortality risk. S2 Fig. Forest plot analysis of NAFLD/MAFLD and all-cause mortality risk. S1 Table. PRISMA checklist. S2 Table. Excluded studies. S3 Table. Newcastle-Ottawa Scale evaluation of included observational studies. S4 Table. Diagnostic criteria for NAFLD and MAFLD in included studies. S5 Table. Search strategy. S6 Table. GRADE assessment. (DOCX) [file pone.0312650.s001.DOCX]
